# Miscellany

**Published:** 1828-04-01

**Authors:** 


### MISCELLANY.

### 1. Carbonate of Potash and Opium, in Traumatic Tetanus.

Many of our readers will remember that, a few weeks ago, Dr. Barry mentioned, in the Westminster Medical Society, a practice which he had tried, with success, in traumatic tetanus, while serving with the Portuguese troops in the Peninsular war. This practice was, *alternate doses of carbonate of soda and opium*. The alternation of these medicines was suggested to him by another gentleman, who probably learnt it in Germany, where it has got the appellation of the "method of Stultz." A case has lately been published in Hufeland's Journal, and copied into the Revue Medicale, which elucidates this practice.

*Case.* A young woman of 19 years of age, robust and healthy, received a contused wound on the instep of the left foot—for which nothing was done by any surgeon. A month after the accident, and before the wound was healed, she was suddenly seized with trismus, which went on to opisthotonos. The physician who was called in, found the patient in this state, the wound in the foot being in a bad condition, and covered with proud flesh. Thirty-two ounces of blood were abstracted—purgatives were given—and poultices were applied to the wound. The bowels being opened, the method of Stultz was put in force, and, in the course of twelve days, she took 224 grains of opium, in alternate doses with carbonate of potash, the quantity of the latter not being mentioned. Under the influence of this treatment, the spasms became less severe and less frequent, till a general swelling of the body came on, when they ceased altogether. This swelling appeared to be of an anasarcous nature, and gave way to diuretics. It was succeeded by an eruption on the skin resembling scarlatina. This last disappeared, and the patient got quite well.

The rationale of this practice, we believe, has been attempted on the supposition that, the alkali enables the opium to get at the nervous surface of the stomach much more readily than it otherwise would do—and that, in short, it is the preparation which gives the latter remedy the complete power of acting. As opium is still the medicine on which reliance is chiefly placed in this terrible disease, and as there appears no counter-indication to the alkali, we think the method should be tried.

### 2. Pathological Investigations in the Dissecting-room.

Not unfrequently bodies are brought into the dissecting-room presenting pathological appearances worthy to be recorded, although, unfortunately, we thus become acquainted only with the phenomena in the dead body, not with the symptoms which indicate them in the living. In the Bibliotheque for November last, M. Cruveilhier has published an account of a dissection, which we shall notice here.

*Varices of the Veins of the Round Ligament.*

It is well known that the veins of the spermatic cord in the male, are subject to a varicose enlargement, and that this affection, has been occasionally mistaken for an inguinal hernia. We remember, a month or two ago, seeing a case at one of our hospitals, where a young man in a drunken fray got a blow on, or sprained his right groin, in which a tumour immediately appeared. Some few hours afterwards he was admitted into the hospital, having all the symptoms of strangulated inguinal hernia, which was treated by the warm bath, bleeding, the taxis, &c. without relief. The operation was fixed for 4 P.M. but in the mean time, cold, by means of the sulphuric æther, was steadily applied. The surgeons and pupils had collected, and all was prepared for the operation, when a portion of gut suddenly " went up" under a moderate application of the taxis. Considerable swelling, however, still remained, and it eventually turned out, that there had been an inguinal hernia, combined with varicocele and hernia humoralis, the patient having laboured under a clap for some time previously.

The patient, we should say the *subject*, which came into the hands of M. Cruveilhier, was a female, about 60 years of age, having an oblong tumour at each external ring, which M. C. imagined to be a brace of herniæ, and proceeded to operate on one of them accordingly. On cutting through the skin, the external pudic veins were found to be very tortuous, and one large vein in particular was seen to cover the upper pillar of the ring. Continuing the incision, the knife came down to a muscular substance, resembling a hypertrophied bladder, or uterus at the 3d month of pregnancy. M. Cruveilhier thought he had come upon a hernia of the bladder, but *n'importe* he cut on, divided the " fleshy tissue" into laminæ, and at last opened into a large vessel, which proved to be a vein with its coats much thickened. The operator was now fairly bothered, and determined to satisfy his doubts by cutting into the abdomen, and introducing his finger from that into the ring. To shorten a long story, it was discovered that the tumour was made up of the " muscular substance" of the round ligament considerably thickened and hypertrophied, and its veins bound together by fibrous bands, and exceedingly varicose. The veins of the legs were very varicose also.

This good lady must have been a museum in herself, for besides the above rare affection of the round ligament, she presented some curious appearances in the abdomen. The mesentery commenced at the pylorus, so that the duodenum was contained within its laminæ, and not distinguishable in this respect from the small intestine generally. The right half of the pancreas was contained in the mesentery, and the cæcum, ascending, transverse, and descending arch, and sigmoid flexure of the colon, formed but one tortuous arch, not above half its usual length. Several other anomalies in the mesentery and epiploon, were observed, but we need not stop to describe them. The lower half of the anterior wall of the vagina was affected with cancerous ulcerations, by which it communicated with the bladder. The base of the ulceration was white, brittle, and prominent, several little prominences projecting into the bladder, and one of them being covered by a calculous deposit of uric acid.

Whilst upon the subject of dissecting-room pathology, we may take notice of a paper published by Mr. Cæsar Hawkins, in No. 10 of the Medical Gazette, containing an account of some appearances observed in two bodies brought into the School of Great Windmill-street. The first was a middle-aged man, who had been affected with ricketts to an extreme degree. It appeared that, whilst labouring under this disease, the left knee had been dislocated, so that the tibia rested on the fore-part of the femur. In this situation, a new joint had been formed, the back part of the head of the tibia being flattened, and a kind of cup having formed on the front of the femur, above the condyles, which was rendered more complete by the growth of a large knob of bone immediately above it. The surfaces of this new articulation were coated with an imperfect cartilage, and provided with a distinct synovial membrane, and ligaments of considerable strength.

The patella of that side was much smaller than the other, probably, as Mr. Hawkins observes, from its not having been called into exercise, the new form of joint not allowing of flexion and extension of the leg. The hip-joints were remarkably altered also, the neck of the femur being on each side absorbed, so that the trochanter and head of the bone were on a level with each other. The head itself was flattened and widened, as was the acetabulum, which was much shallower than usual. From these circumstances, Mr. Hawkins thinks it is evident that the enarthrotic, or ball and socket articulation was lost, and had degenerated into little more than a mere hinge-joint. The patient had been obliged to use crutches, so that the head of the humerus was forced up against the acromion, and between these parts there was found a number of enlarged bursæ, filled with a quantity of thick gelatinous fluid. Thus, the very pressure and irritation produced their remedy, namely, the interposition of these additional friction-wheels between the ends of bone.

In the second case detailed by Mr. H. it appears that the median nerve, flexor digitorum sublimis, and flexor carpi radialis muscles, and the radial artery, had been divided by some wound inflicted a considerable time previous to the patient's death. The portions of the flexor digitorum were united by an imperfect ligamentous substance, and the flexor carpi was partly deficient. The radial artery was likewise deficient for nearly three inches, its lower part being supplied by an enlarged branch of the interosseous. But the more interesting appearances were observed in the median nerve. This terminated, about the middle of the fore-arm, in an oblong tumour of a light brown colour and firm consistence. The filaments of the nerve were separated, and spread out over its upper end, whilst at its lower, the tumour had contracted inseparable adhesions to the ligamentous part of the flexor sublimis. The nerve below the annular ligament was perfectly natural, but between this lower portion and the tumour on the upper, there was a separation of at least three inches, without any intervening substance whatever. An enlarged branch, however, was given off from the superficial division of the muscular spiral, which passed through the flexor sublimis and joined the lower portion of the median nerve. One or two smaller anastomoses were also observable between these nerves nearer the wrist.

---

### 3. Extract of Valerian, in full doses.

Every practitioner is aware that valerian exerts a very considerable sedative influence over the nervous system; but the extremely disagreeable odour of that root (to some more offensive than assafœtida) very much limits its use, especially among females, where it is most wanted. Dr. Guibert, of Paris, has recently exhibited the extract of valerian, in doses varying from one to two or three drachms per diem, in several disorders of the nervous system—and, if the cases detailed be faithful, with considerable success. As the medicine is perfectly safe—as the root has long been employed with advantage—as its bulk and flavour are objectionable—and as the extract appears to possess all the medicinal properties of the root, divested of several inconveniences, we think it is a medicine which deserves attention, particularly in a number of female complaints equally distressing and unmanageable.

The cases in which Dr. Guibert has employed the extract are spasmodic contractions of the muscles of the limbs—nervous tremors in adults, when unaccompanied by any signs of plethora—chorea—epilepsy, in which disease he has found the valerian very serviceable, after sanguineous and other evacuations—palpitation of the heart, when of a nervous character, as they very often are—spasmodic asthma, and nervous dyspnœa—hooping cough, in its later stages, and after the inflammatory symptoms have subsided—dyspepsia and gastrodynia unattended with any obvious inflammatory symptoms—vomitings of a nervous character, and not apparently dependent on any organic disease—and last, not least, the various proteian forms of hysteria. Of all these complaints, the Doctor has adduced examples in our Parisian cotemporary—the Revue Medicale, for December last. These cases we shall not detail; but we think the remedy is worthy of the English practitioner's notice.

### 4. Diabetes Mellitus.

A late meeting of the Westminster Medical Society was occupied with the subject of diabetes. Dr. Ayre (to use the learned language of the Lancet) "*prefaced* the relation of a case with a *Peroration*," which we would call an *exordium*, on the pathology of the disease. The Doctor did not agree with Heberden, that it was a breaking up of the constitution in advanced life—nor with Cullen, that it was nearly always fatal—nor with those who considered it to be dependent on a faulty digestion,—an imperfect assimilation,—or a bad sanguification. In short, he looked upon diabetes to be a local disease or disorder of the kidneys—the nature of which, he thought, was chronic inflammation. Entertaining this view, he therefore expected but little from any peculiar diet tending to prevent the formation of saccharine matter in the fluids, and its elimination by the kidneys. Local detractions of blood from the loins formed almost the whole of his therapeutics. He stated the details of a case, part of which had been drawn up by the patient himself, from which it appeared that the quantity of diabetic urine amounted, at one time, to more than 20 pints per diem, while the thirst was unquenchable, and the appetite voracious. The plan of treatment abovementioned was put in force, and by the time the patient had been seven times cupped on the loins, the disease was subdued (or supposed to be so) and the health restored. Unfortunately for the efficacy of this plan, Dr. Barry informed the Society that the same patient had come under his care, shortly after he left Dr. Ayre, and the diabetes had returned nearly as bad as before. Dr. Barry had put the gentleman on Rollo's plan of animal food diet, and to this the disease was once more giving way.* A sharp discussion now took place respecting the chemical pathology of diabetes—Dr. Barry maintaining that the grand source of the malady would be found in the fluids, while Dr. Ayre, Mr. North, and some others, considered the solids in fault. Mr. North brought forward the authority of Berzelius to prove that, in certain affections of the liver, the urea disappears from the urine, and a saccharine principle is there evolved—in short, diabetes produced.

Dr. Johnson was inclined to agree with Dr. Barry, that the pathology of diabetes could not be affixed on any one particular organ—neither the kidneys, the liver, the stomach, or the lungs, exclusively. In all the severe cases which he had seen, however, there was some evident functional disorder of the system accompanying the diabetes. In every case where he had an opportunity of examining or seeing examined, the body after death, the lungs were found affected with disease, whatever were the appearances in other organs. In some researches which he made among the most authentic dissections that have been recorded of this disease, he found that, in very few instances indeed, were the lungs in a state of integrity. By this he did not mean to infer that disease of the lungs was the cause of diabetes; but he thought that this fact authorised us to suspect a considerable connexion between disordered function in the respiratory apparatus, and the production of diabetic urine. In respect to treatment, he had found restriction to animal diet, with occasional blood-letting, and great attention to the improvement of such functions as appeared to be deranged, the most successful plan. The general sense of the Society appeared to be in favour of the mode of treatment recommended by Rollo. Dr. Ayre adhered to his opinion, that the depletive plan of Watt, was superior—and especially local depletion, from the neighbourhood of the kidneys. It appeared, indeed, that, in the case detailed by Dr. Ayre, even admitting that the result was not conclusive, the cupping on the loins always gave temporary relief. This the patient himself acknowledged to Dr. Barry, though he got tired, he said, of the plan, as the relief was transient.

---

### 5. Surgical Curriculum.

In our last Fasciculus we intimated our intention of examining seriatim, the different items in the recent curriculum of

* This discrepancy was afterwards satisfactorily explained.—*Ed.*

the College of Surgeons. Entertaining, as we do, the greatest respect, and even friendship, for several individual members of the Council, and knowing that many of them are anxious to do every thing that is liberal and conducive to the public good, we cannot but hope that the observations of those who must be disinterested on the subject will be taken in good part, and not attributed to sinister motives, or a disposition to hypercritical censure. In our remarks, we shall appeal entirely to the judgment, and not to the prejudices, of all concerned. In the present instance, we shall limit our inquiry to the first clause of the curriculum.

1. "THE ONLY SCHOOLS OF ANATOMY AND PHYSIOLOGY RECOGNIZED, ARE, LONDON, DUBLIN, EDINBURGH, GLASGOW, AND ABERDEEN."

We should have thought that the liberal opinions lately introduced by many—indeed we might say by all, the most talented and enlightened men of the age, in respect to free trade, and international reciprocities of interest, would have operated on the minds of men of science and literature, and that *they* would not have enlisted themselves on the side of ignorance, narrow-minded policy, John-Bull prejudice, and downright opposition to the advancement of that science and literature, of which they are, at once, the guardians and the ornaments. And, of all branches of human knowledge, that of medicine and surgery should surely be the most entirely emancipated from local, or even national restrictions, seeing that it is the same in kind, on every point of this great globe which we inhabit. We have no objection to that patriotism which aims at raising the character and acquirements of one nation over those of other nations. This is laudable, and tends to useful emulation among all. But we most decidedly object to that principle which limits or impedes the *means* of acquiring that knowledge which raises the character of individuals, and consequently of nations. What would be thought of the Russian Autocrat, if he threw every kind of impediment in the way of his naval, military, and medical subjects, who sought to improve themselves in ship-building, military discipline, and the practice of physic and surgery, by sojourning in Paris, London, or Vienna? We would call him a Goth, a Tartar, or a Vandal! But he has not permitted us to accuse him of such barbarism. He has encouraged every individual exertion of this kind—not only by withdrawing every obstruction, but by holding out positive rewards. Yet *we* consider all medical knowledge as limited to the soil of Britain—and instead of fostering the praiseworthy endeavours of individuals to draw professional lore from every spot where it can be procured, we issue our little injunctions against all science that is not taught on half a dozen points or dots on the surface of the British Isles! How is this reconcileable with sound sense? How does it quadrate with a maxim as old or older than the Christian æra—"fas est et ab hoste *doceri*." When we reflect on the liberality, the enlightened policy of the great medical schools of Europe, where English study is allowed to form part of the curriculum, we blush for the institutions of our own country, which enact statutes that would do no credit to the intellects of a Cossack, an Otaheitian, or Canadian! We know, indeed, that there are men, connected with our chartered bodies, who have the effrontery to stand up, even in our medical societies, and whine forth, with methodistical cadences, their little interested orations about the excellence of medical jurisprudence in this country, and the complete superiority of British *pathology* over that of every other people in Europe. They are aware that this flattering unction will gain the smile of the moment, from those who are engaged in the same line of policy as themselves; but, as long as our hand can wield the pen, we will bring this "mentis gratissimus error," before the bar of public reason, for adjudication.

What is the collegiate excuse for this restriction of anatomical and pathological study to certain schools in this country? The old corn-law policy. Favour the English farmer. Protect the farmers of anatomy and surgery in Guy's and Bartholomew's, although the consumers, the students, may pay twelve or fifteen guineas for each body, and consequently may be half-starved during each season! But why do the anatomical farmers import their wine from the Bordelais, when grapes are seen mantling on the walls of their country-houses? Because the vine grows plentifully in the fields there, and can only be trained on the sunny sides of their houses here. It is the same with anatomy. The dear and scanty supply in our dissecting rooms must be put up with, and preferred to, the cheap and plentiful supply in Paris—and all this for the encouragement of the British anatomical farmer! The illiberality of this policy is undeniable, and what are the advantages, after all, which the monopolists can hope to gain by it? There is not one in one hundred of English students who has the means of travelling to other countries for anatomical, pathological, or clinical improvement. Yet to deter this one in two or three hundred, from prosecuting a portion of his studies in those places which are peculiarly favourable, the council of the College has enacted a law which must be viewed by every enlightened man in Europe as a libel on the English profession! The sting of this libel, too, is rendered the more galling, when we see such a place as Aberdeen "recognized," while Paris is "incognizable!" Was it possible that the members of the Council could preserve their gravity when they put their seal to this primary and fundamental law? What reason has been assigned for giving the prerogative to Aberdeen, in preference to twenty other and better places? Because, forsooth, there is an anatomical CHAIR in Aberdeen! Why so there is in Oxford and Cambridge, and yet we do not see these "venerable seats of learning," recognized by the College. Now anatomy and pathology may be *taught* by a chair or by any other wooden machine—but it can only be *learnt* by dissection. Dissection cannot be performed without bodies—and bodies cannot be procured, except where there is an abundant population and consequent mortality. If these be axioms, and we believe they are, the statute which recognizes Aberdeen, as a school of anatomy and physiology, and rejects Manchester, Liverpool, and other great provincial cities and towns, where hospitals abound, bodies can be procured, and excellent anatomists reside—is a blot on the justice and common sense of the country. The sooner the College revise and amend this first item in their curriculum, the better. It would be only decency as well as liberality to admit one year, at least, of Parisian or other continental time in the curriculum—it would be good policy, inasmuch as it would hold out some little encouragement for young men to extend their knowledge and expand their minds, by visiting the institutions of foreign countries, where clinical practice is open to strangers, and anatomy can be prosecuted at a very trifling expense, instead of binding them down to study anatomy, where dissection is either impracticable for want of subjects, or ruinous by the expense of them. So much for the first count in the indictment. We shall discuss the other counts in succeeding fasciculi of this Journal.

---

### 6. INGUINAL ANEURISM—EXTERNAL ILIAC ARTERY TIED. By MR. BRODIE.

It is well known how very successful the operation of tying the external iliac has proved in the hands of surgeons, both in this country and abroad. It is to Mr. Abernethy that we are indebted for it, and really the history of the successive attempts made by that gentlemen reflects the highest credit upon his firmness and abilities. At the time Mr. Hodgson's valuable Treatise on Aneurism was first published, namely, in 1815, twenty-two cases had occurred, of which fifteen had proved successful. Since that period the operation has been frequently repeated, and, upon the whole, with great success, so much indeed, that it has been proposed to have recourse to it in popliteal aneurism, a plan which is not very likely to be put in practice, or, if put in practice, to be of service.

The subject of the present case, which we had an opportunity of witnessing, and shall detail very briefly, was a tailor, æt. 38, who had been exposed to damp and cold in White-Cross Prison, and had suffered a great deal of anxiety on account of his family. In November last, whilst having connexion with his wife, he felt as if something had given way in the left groin, and soon afterwards noticed a small throbbing tumour in the part. It increased gradually for a fortnight, then remained stationary until the latter end of January, when it again began to swell, and the limb became œdematous. When admitted into St. George's, February

15th, the aneurismal tumour was large, apparently extending the whole length of the common femoral artery; rather triangular in form, occupying the triangular space between the sartorius and pectinæus; reducible, but not *removeable* by pressure; bounded abruptly above by Poupart's ligament; and pulsating very forcibly. Pressure upon the external iliac arrested the pulsation, and diminished the tumour, whilst pressure on the superficial femoral below* *lessened* the pulsation, but had little or no effect upon the tumour. The whole limb was swollen and tense, the foot œdematous and numb. There was much pain in the direction of the external branches of the crural nerve; the countenance was sallow and anxious; the appetite indifferent; tongue red; bowels costive; pulse quick and hard. Under these circumstances, he was placed in bed, bled once or twice, and purged, until the 21st, when, being in a more favourable state for the operation, and rather anxious for its performance, the external iliac was tied by Mr. Brodie. We were present at the operation, and must say that it was performed with a great deal of coolness and facility. The plan adopted, was that of Mr. Abernethy's, with some little modifications, and the artery was readily discovered and secured, but, in consequence of the vessel having contracted some adhesions to the surrounding parts, care was required in passing the ligature around it. A thin silk thread was employed, one end cut rather short, and the other brought out at the external wound, which was brought together by sutures and adhesive straps. In the evening, the pulse had got up a little, and at 12 P.M. next day, he was labouring under the following symptoms. Countenance anxious—breathing hurried and difficult—pain in the right side, increased on making a deep inspiration, speaking loud, or coughing—pulse corded and full—surface covered with perspiration—tongue white and thickly coated—thirst—confined bowels. He was bled to oz. xviij. with the effect of relieving the pain and diminishing the anxiety of countenance. He was likewise directed to to take a senna draught every six hours until it operated. From this time no unfavourable symptoms had occurred up to the 28th, when we saw the patient. He still laboured under some cough, but it was unattended with pain in the side, or hardness of pulse. The countenance had lost the anxiety which marked it prior to the performance of the operation—the pain in the thigh had entirely disappeared—the swelling of the limb was subsiding, and the wound was going on very favourably. It may be mentioned, that on drawing the ligature, the pulsation in the tumour ceased, and never afterwards returned, but the tumour did not very materially diminish in size at the time. Since that, however, it has gone on steadily decreasing and a pulsation is distinguishable in the superficial femoral, as well as in another branch on the inside of the knee, probably the anastomotica magna.

It will be observed that Mr. Brodie adopted Mr. Abernethy's mode of operating, in preference to Sir Astley Cooper's. In a clinical lecture on the case, Mr. B. assigned as a reason, that he had twice done the former with success, and therefore was more *au fait* at it, and that as he had reason to believe the lower portion of the external iliac implicated in the disease, he naturally adopted that mode of operating, which would enable him to arrive at the upper portion of the artery. There is another disadvantage attending Sir A. Cooper's method (viz. a semi-circular incision carried almost transversely beneath the internal ring) which is this, that there is a danger of wounding the epigastric artery. This accident happened to no less a personage than the French "Leviathan of Surgery" M. Dupuytren, in 1821, and the patient died of peritoneal inflammation. These were some of the reasons assigned by Mr. B. for operating as he did, but there was yet one more, and that a forcible one. In the low, or Sir A. Cooper's, operation, the ligature is applied just above the giving off of the epigastric and circumflexa ilii, and the consequence is, that the plug may be so

* Mr. Hodgson, in his Treatise, asserts that pressure on the artery *below* renders the tumour more tense, and the pulsation more *violent*; indeed, he recommends this as a diagnostic of aneurism. From this report, for the correctness of which we pledge ourselves, it is evident that Mr. Hodgson made too sweeping a statement.

inconsiderable between the ligature and these vessels, as to afford no sufficient security against secondary hæmorrhage. This is no visionary objection, for in a case in which M. Dupuytren tied the vessel at this situation, in the Hôtel Dieu, the patient nearly sank under secondary hæmorrhage evidently proceeding from the lower portion of the artery. On examination, M. D. perceived the epigastric to have become enormously enlarged, and to have brought the blood round almost to the verge of the ligature, on the separation of which, the adhesion between the inner coats of the vessel gave way, and bleeding was the result. In another instance, Sir A. Cooper tied the *femoral* artery, immediately *below* the origin of these branches, the epigastric and circumflexa ilii, and in that case also secondary hæmorrhage followed. The same thing, we believe, happened to Mr. Cline; and in the case at St. George's Hospital, upon which so much valuable indignation has been lately thrown away, the ligature is said to have been applied a little below the giving off of the profunda, and it is not unreasonable to conclude that this circumstance favoured, if it did not actually occasion, the occurrence of the secondary hæmorrhage.

In the report it will be observed, that the subsidence of the tumour did not immediately follow the application of the ligature, and this, we believe, is always the case in what Scarpa calls " false aneurism." In the " true aneurism," which is merely a dilatation of the vessel, and which if let alone, will very often remain stationary for a considerable length of time, or even during the patient's life, if it be an old person, the contrary is the case, the tumour generally collapsing remarkably after tying the vessel. This, we apprehend, will afford a key to several circumstances connected with some of those operations on the arteries, which have lately astonished the surgical world.

The bleeding practised in this case on the day after the operation, was bold and decisive, and was, in all probability, the means of saving the patient's life. The pleuritic affection with which he was attacked was insidious, but not the less dangerous, and had it not been " put out" at once, as it was by the decisive blood-letting, it might soon have proved more than a match for the lancet, or any other remedy. These inflammations of the pleura, all know, are not uncommon after severe injuries or operations, and in the case of ligature of the carotid, recorded in our third fasciculus, it carried off the patient in a very days.

We see nothing in the details of the case which would lead to an unfavourable prognosis. The ligature is yet to come away, but there is no good reason, which we are aware of, to apprehend secondary hæmorrage on its separation. However, as the French say—nous verrons.

---

### 7. WOUND OF THE KNEE—EXTENSIVE SUPPURATION—AMPUTATION OF THE LIMB.*

This case occured under Mr. Travers, and, as far as we can understand it, (which, from the manner in which it is drawn up, is no very easy matter) the particulars are as follow.

The patient, æt. 52, of "*lax fibre*" and intemperate habits, received a lacerated wound over the patella, denuding the bone, but not opening into the knee-joint. This was on the 23rd, and on the 26th Dec. the wound was granulating, and all was well, save that at the lower part of the patella, there was " a dark coloured slough, the size of a shilling, *partially* adherent." He was ordered quinine, porter, and diluted nitric acid lotion to the part, and the next date of report is Jan. 30th, upwards of a month. At this time the slough had *partially* separated, so that it must have been almost in *statu quo;* the bone was exposed; there was severe pain in the joint, and on pressing the side of the wound, fetid pus escaped. Pulse small and quick—bowels costive. Wine and opium were administered, but an opening formed on the outer side of the joint, leading into its cavity, and discharging a quantity of dark-coloured, offensive pus, and on the 5th Feb. the leg and foot were œdematous—the integuments inflamed and painful—discharge from the joint profuse—and the pulse small, weak, and innumerably rapid. These symptoms are noted down with the most careful ambiguity, but still they seem to

* Lancet, No. 234.

furnish an idea, faint and glimmering we own, of the characters of an affection, termed by nosologists erysipelas; at any rate, if the œdema, and inflammation of the *integuments* did not depend on erysipelas, we confess that we are totally unable to divine what they *did* depend upon. Under these circumstances, amputation of the thigh was proposed, and performed by Mr. Travers, at its middle, in consequence of the "cutaneous inflammation" having extended to its lower part. On examining the amputated limb, the leg and foot were found enormously enlarged,—a large gangrenous patch, surrounded by a livid redness, occupied the calf, and there were several vesicles at the upper part of the leg. On making an incision into the limb, a mixture of *pus* and serum, escaped from the subcutaneous cellular tissue, as well as from that connecting the various muscles. The joint contained much offensive pus—its synovial membrane was highly inflamed—its cartilages softened, and in one part ulcerated, and the patella was softened, and "partially detached." The patient, on the second day after the operation, became delirious—there was pain in, and profuse discharge from the stump, which was of a dirty ash-colour, and, on the morning of the 11th, he sank.

This report is so confused, and the language so unlike what is commonly known as the King's English, that we cannot be at all sure of the exact nature of the case;—as far, however, as we can judge, it appears to have been an attack of "gangrenous erysipelas" occurring, as that terrible disease generally does occur, in a person of weakened frame, and intemperate habits. Whether the suppuration within the joint, preceded, accompanied, or succeeded the erysipelatous inflammation, is not particularly clear, but, whichever was the case, it is obvious that amputation was, indeed, a *dernier resort*, and, moreover, a very hopeless one.

---

### 8. Curious Specimen of Ratiocination.

The Profession is well aware that the Lancet has plumed itself for the illumination which it has been able to throw on the non-professional public, and on the jury in Mr. Stanley's case, by which the said non-professional public are taught to place no reliance whatever on the professional skill or veracity of a Cooper, a Brodie, a Bell, a Travers, an Abernethy, &c. By proving that such men are ignorant, the Lancet sagaciously concludes that the public will, at once, decide that surgeons in general are infallible—that, in fact, the detection of an error in the *former* class will for ever absolve the *latter* from all suspicion of such weakness!

"As to the *effect* (says the Lancet) produced by this trial on the minds of the public, it is decidedly favourable to the great body of the Profession. It is calculated to raise the great mass of English surgeons, *absurdly* denominated general practitioners, in public estimation."*

This specimen of reasoning has no parallel in the annals of medical literature! Those men who have arrived at universal eminence in their profession—by whom a great portion of the Profession has been taught—whose opinions are sought after and respected, both by medical men and by the public at large—these men, we say, are to be held up to ridicule, as totally ignorant—and, as a natural corollary, all those who have derived their opinions and practical precepts from these men, must rise in public estimation! One would really suppose that the Lancet considers its readers as totally incapable of a single exertion of

---

* We would ask the Lancet where is the absurdity, or the degradation of the term "General Practitioner?" It is that under which Hippocrates, and some of the most eminent in our profession have practised. It supposes a general acquaintance with all branches of the Profession—and without which acquaintance, no surgeon, no physician can be a good practitioner. This sentence of the Lancet is the greatest libel ever penned against the "General Practitioner." The Lancet is for ever running out against "pure surgeons," and now it turns round and ridicules the term "General Practitioner," by which it must advise the change to "*pure* surgery;" such as it is practised by Thomas Wakley, William Lawrence, and James Wardrop.

thought—as completely devoid of every particle of intellect! The conclusion which the PUBLIC must draw from all this (as far as the public can be influenced by such a tool as the Lancet) is this—that we are, one and all, from the highest to the lowest in the Profession, a set of block-heads—ignorant of our art—and leagued to screen each other when detected! Among practitioners, we have not heard a single dissentient voice as to this *effect* which the writings of the Lancet are calculated to produce. But we shall come a little closer to the point, and prove to a demonstration, the inconsequences of the Lancet's reasoning. This instrument; this "scarificator," in its rage to excoriate an hospital surgeon, has trod under foot and traduced three General Practitioners—and then, with unparalleled effrontery, tells these last, that the whole procedure is decidedly favourable to that class of the profession to which they belong! First there was Mr. Jarman, who saw Mr. Rolfe immediately after the accident—who washed away the gravel—who found a "hard moveable substance in the interior, lying about an inch from the knee-pan"—who attended the patient home, helped Mr. Stanley to examine the limb, and applied himself the splints.

Now we do not attach the slightest blame to Mr. Jarman for not discovering the true nature of the accident—but, in the name of justice, is he not as much responsible for the mistake as Mr. Stanley—must not all censures levelled by the Lancet against the one, fall equally on the other, in the minds of the public? Then, again, there was Mr. Janet, whom we happen to know, and who is an experienced and able general practitioner. He attended with Mr. Stanley—but he could not ascertain the precise nature of the hard substance near the patella, and he, too, is included in the sweeping accusations brought against the hospital surgeon. We now come to Mr. Lilly. This gentleman candidly acknowledged in court, that, after several examinations—"he felt the hard substance, and thought it was bone."—*Lancet, No.* 234, *p.* 746. It is true that Mr. Lilly discovered his mistake, when the flint perforated the skin, and displayed its real character; but surely there was nothing in this to ground a contrast between his judgment and that of Mr. Stanley. Thus, then, we see three GENERAL PRACTITIONERS, and one HOSPITAL SURGEON, all involved in one general charge of ignorance or incapacity, (for where all were concerned in the case, and neither of them discovered the stone till it partly came through the skin, all are necessarily included in the accusation, whether ill or well founded) and yet this instrument of justice insults the understandings of its readers, by asserting that, in damning one out of the four, the other three must be benefited!

An impartial examination of the evidence, then, as well of the general practitioners who actually saw the case, as the distinguished surgeons who heard of it, leads us to infer, that the obscurity of the accident would have prevented the generality of surgeons, of whatever denomination, from ascertaining, with any degree of certainty, the true nature of the case, before the flint made its way through the skin.* Two of the medical attendants (Mr. Stanley and Mr. Janet) we know personally and professionally; and, at the risk of another action for libel, we declare our firm conviction, that they are not inferior to that celebrated, experienced, and successful surgeon, Thomas Wakley himself, by whose advice, in opposition to that of Sir Astley Cooper, Mr. Lambert performed his renowned operation, now making the tour of Europe, and which enabled the said Mr. Lambert (ultimately) to remove, without pain or hæmorrhage, several inches of a troublesome and misshapen carotid, as thousands of passengers along the Strand can verify by oath. We have now, we think, proved, to a demonstration, that the attempt to draw public odium on Mr. Stanley, must in-

* Had we been called on for an opinion upon oath, the above is that which we would have given. A mistake was made by all concerned in this case, as the event shewed—and the probability is, that the same mistake would have been made by others—even by those who so loudly cry out shame! We neither censure nor applaud the evidence given by the hospital surgeons on this trial—we only state what we would ourselves have offered. It is probable that a more moderate and diffident tone in the witnesses would have been better for the defendant.

evitably draw public odium on all classes of the profession, not even exempting that class which has the misfortune of being selected by the most scurrilous publication that ever issued from the press, as the peculiar object of its mean sycophancy and nauseating adulation. That class stands not in need of such an advocate as the Lancet—whose censure is applause—whose praise is poison!

---

### VARIETIES.

### 1. AMMONIACAL SULPHATE OF COPPER IN EPILEPSY.

In a late Number of Hufeland's Journal, Dr. Urban, of Bernstadt, has published some cases of epilepsy, cured by the above medicine, which, indeed, is not a new remedy. It is to be remembered, however, that Dr. Urban confines this treatment to those who evince no corporeal lesion, as the cause of the disease, and that he puts his patients on a very strict regimen, employing proper evacuations, sanguineous and intestinal, from time to time. These means are, perhaps, not merely auxiliaries, but principals in the methodus medendi.

### 2. MURIATE OF IRON IN SOFTENING OF THE STOMACHS OF CHILDREN.

Dr. Pommer having lost two children affected with vomiting and purging, found, on examination, that the mucous membrane of the stomach was in a state of ramollissement, or softening. He accordingly treated some other infants similarly affected, with the hydrochlorate of iron, and saved them. He was induced to employ this remedy from the recommendation of Professor Autenrieth, who found it very efficacious in arresting the distressing diarrhœas that accompany or supervene on typhus and other bad fevers. We observe, however, that Dr. Pommer has conjoined other means which may have borne a considerable share in the cure. Thus, when the vomiting and purging were severe, he abstracted almost every kind of food and drink, except a few spoonfuls of warm milk twice a day. There being fever present, he applied cold to the head—sinapisms to the legs, and cataplasms to the stomach. After these means, or with them, he employed the hydro-chlorate of iron, and the children recovered. Some cases are detailed in the Heidelberg Annals, which appear to shew the utility of the medicine.

### 3. LIGATURE OF ARTERIES ULTRA TUMORES.

In the ARCHIVES GENERALES for November last, there is given an account of the wonderful success of these operations in this country,—among others Mr. Lambert's case, which is introduced to our Continental brethren in the following terms:—"L'example de WARDROP a été suivi *avec beaucoup de succés*, dans un autre cas d'anevrysme, par le DOCTEUR JAMES LAMBERT." Mr. Lambert's case finishes, in the continental records, most successfully. "La tumeur finit aussi par disparaitre, &c." In the German Journals, all these cases have been published as attended with the most happy results, and German surgeons are called upon to imitate the wonderful exploits of their English brethren! There was a saying, in the olden time, "omnia vincit VERITAS, et prevalebit." We begin to think that the ancients were entirely mistaken, and that the adage should have run thus: "omnia vincit MENDACIUM, et prevalebit." There was a golden age—and there was an iron age—the present appears to be the age of BRASS.

### 4. FATAL HÆMORRHAGE FROM LEECH-BITES.

M. Lisfranc has lately related a case in the Royal Academy of Medicine, which occurred at LA PITIÉ. A female was received into the Hospital with an ulcer, and some symptoms of gastric inflammation. Thirty leeches were applied to the epigastrium, which bled moderately, and then stopped. Three days afterwards the leech-bites opened again, in the middle of the night, and the patient was found dead the next morning, from the profuseness of the hæmorrhage.

